# Paclitaxel-coated balloon fistuloplasty versus plain balloon fistuloplasty only to preserve the patency of arteriovenous fistulae used for haemodialysis (PAVE): study protocol for a randomised controlled trial

**DOI:** 10.1186/s13063-016-1372-7

**Published:** 2016-05-12

**Authors:** Narayan Karunanithy, Irene Rebollo Mesa, Anthony Dorling, Francis Calder, Konstantinos Katsanos, Vikki Semik, Emily Robinson, Janet Peacock, Neelanjan Das, Colin Forman, Sarah Lawman, Kate Steiner, C Jason Wilkins, Michael G Robson

**Affiliations:** Department of Interventional Radiology, Guy’s and St Thomas’ NHS Trust, Guy’s Hospital, Great Maze Pond, London, SE1 9RT UK; Biostatistics Department, King’s College London, London, SE5 8AF UK; MRC Centre for Transplantation, King’s College London, Guy’s Hospital, Great Maze Pond, London, SE1 9RT UK; Renal, Urology and Transplantation Directorate, Guy’s and St Thomas’ NHS Trust, Guy’s Hospital, Great Maze Pond, London, SE1 9RT UK; Division of Health and Social Care Research, King’s College London, Capital House, 42 Weston Street, London, SE1 3QD UK; Department of Interventional Radiology, East Kent Hospitals NHS Trust, Kent and Canterbury Hospital, Ethelbert Road, Canterbury, Kent CT1 3NG UK; Renal Services, Royal Free London NHS Trust, Pond Street, London, NW3 2QG UK; Sussex Kidney Unit, Brighton and Sussex University Hospitals NHS Trust, Royal Sussex County Hospital, Eastern Road, Brighton, BN2 5BE UK; Department of Interventional Radiology, East and North Hertfordshire NHS Trust, Lister Hospital, Coreys Mill Lane, Stevenage, Herts, SG1 4AB UK; Department of Interventional Radiology, Kings’ College Hospital NHS Trust, Denmark Hill, London, SE5 9RS UK

**Keywords:** Arteriovenous fistula, Haemodialysis, Fistuloplasty, Angioplasty, Paclitaxel, Stenosis, Balloon, Neointimal hyperplasia, Clinical trial, Randomised controlled, EME-funded, NIHR, Kidney, Renal, Efficacy

## Abstract

**Background:**

The initial therapy for a stenosis in an arteriovenous fistula used for haemodialysis is radiological balloon dilatation or angioplasty. The benefit of angioplasty is often short-lived, intervention-free survival is reported to be 40–50 % at 1 year. Previous small studies and observational data suggest that paclitaxel-coated balloons may be of benefit in improving outcomes after fistuloplasty of stenotic arteriovenous fistulae.

**Methods/design:**

We have designed a multicentre, double-blind randomised controlled trial to test the superiority of paclitaxel-coated balloons for preventing restenosis after fistuloplasty in patients with a native arteriovenous fistula. Two hundred and eleven patients will be followed up for a minimum of 1 year. Inclusion criteria include a clinical indication for a fistuloplasty, an access circuit that is free of synthetic graft material or stents, and a residual stenosis of 30 % or less after plain balloon fistuloplasty. Exclusion criteria include a synchronous venous lesion in the same access circuit, location of the stenosis central to the thoracic inlet or a thrombosed access circuit at the time of treatment. The primary endpoint is time to end of target lesion primary patency. This is defined as a clinically-driven radiological or surgical re-intervention at the treatment segment, thrombosis that includes the treatment segment, or abandonment of the access circuit due to an inability to re-treat the treatment segment. Secondary endpoints include angiographic late lumen loss, time to end of access circuit cumulative patency, the total number of interventions, and quality of life. The trial is funded by the National Institute for Health Research.

**Discussion:**

We anticipate that this trial will provide rigorous data that will determine the efficacy of additional paclitaxel-coated balloon fistuloplasty versus plain balloon fistuloplasty only to preserve the patency of arteriovenous fistulae used for haemodialysis.

**Trial registration:**

ISRCTN14284759. Registered on 28 October 2015.

## Background

The 2012 UK Renal Registry report (www.renalreg.com) found that 43.9 % of patients with end-stage kidney disease in the UK are on haemodialysis. This equated to 365 patients per million population in the UK in 2011. This number has increased every year with an overall increase of 3.6 % from 2006 to 2011. In order to perform haemodialysis, reliable vascular access is essential. It is universally agreed that native arteriovenous fistulae (AVF) are superior to synthetic arteriovenous grafts (AVGs) and tunnelled central venous catheters for haemodialysis access. AVFs and AVGs have limited lifespans. Data from the Dialysis Outcomes and Practice Study (DOPPS) showed that in the US the 1-year patency for AVFs and AVGs are 68 % and 49 % respectively. In Europe, 1-year AVF survival was somewhat better at 83 % but there is still a need for improvement [1].

Problems with vascular access are an important cause of morbidity and mortality in haemodialysis patients. In the US, it has been estimated that $1 billion per year is spent on vascular access and its complications [2]. A recent survey in the UK found that haemodialysis patients occupy 320,000 bed days per year, with 30 % of admissions related to vascular access (Renal Association vascular access audit, available at www.renal.org). When thrombosis or stenosis occurs in an AVF or AVG, a central venous catheter may be used for several months until another AVF or AVG is formed and becomes usable. Data from the US have shown that the risk of invasive infection is increased 100-fold in haemodialysis patients compared to the general population. Eighty-five percent of those diagnosed with an infection have an invasive device in situ. Ninety percent of those diagnosed with an infection require hospitalisation and there is a 17 % associated mortality [3]. It is, therefore, imperative to preserve each AVF or AVG for as long as possible and to minimise the use of central venous catheters.

The initial therapy for a stenosis in an AVF is radiological fistuloplasty. A major concern, however, is the longevity of this effect. Turmel-Rodrigues et al. reported the outcomes of interventional salvage of dysfunctional and thrombosed haemodialysis circuits [4]. There were 220 cases in the dysfunctional AVF group. The 6-, 12-, and 24-month primary patency (AVF working with no repeat intervention) reported rates were 67 %, 51 % and 37 % for forearm AVF and 57 %, 35 % and 24 % for upper arm AVF respectively. More recently Bountouris et al. reported the outcomes after 159 percutaneous transluminal angioplasties (PTAs) in AVFs. The primary patency rates at 6, 12 and 24 months were 61 %, 42 % and 35 % respectively [5]. Primary assisted patency rates (AVF working regardless of repeat intervention) were 89 % and 85 % at 6 and 12 months respectively. Although there have been some exceptions [6, 7], most other studies have reported similar primary patency rates of around 40–50 % at 1 year [8–10]. Hence, more durable interventions are required to reduce restenosis rates.

In addition there is a need to better understand the cellular processes involved in the development of stenoses and the responses that occur following intervention. Neointimal hyperplasia is characterised by expansion of alpha smooth muscle actin-positive myofibroblasts in the neointima which leads to stenoses in the venous segments of AVFs [11]. In arteries, the contribution of bone marrow-derived cells to tissue repair depends on the nature and severity of injury [12]. The contribution of bone marrow cells to venous neointimal hyperplasia is not resolved and the data from animal studies are conflicting. Two studies using bone marrow transplantation with cells containing a green fluorescent protein (GFP) or β-galactosidase reporter gene, have suggested a minimal contribution of bone marrow-derived cells in mouse and rat models respectively [13, 14]. However, a further study employing a murine vein graft has suggested that at least 20 % of neointimal cells may be bone marrow-derived [15]. GFP-positive cells were detected by a more sensitive polymerase chain reaction (PCR) method and these technical differences were suggested as a reason for discrepancies with other studies.

In addition to these conflicting data on the origin of neointimal cells, it should be noted that none of the previous reports induced vein injury in a way that mirrors the changes induced by angioplasty. Instead, most have focussed on the development of primary stenosis in venous conduits undergoing arterialisation where endothelium is ‘traumatised’ or activated by changes in the flow characteristics of the arterial blood to which it becomes exposed. Given the data from arterial studies, a contribution from bone marrow cells to the alpha smooth muscle actin-producing cells in the hyperplastic neointima of a dysfunctional arteriovenous fistula is highly likely with the degree of trauma to the endothelium that would follow angioplasty. Angioplasty causes vessel wall damage with rupture of the junction between the intima and the media, with a burst of proliferation and repair. Much of our understanding of aggressive neointimal formation in this context comes from arterial studies [16], but similar pathology and an increase in proliferation has been shown in AVFs following venous angioplasty [17].

Paclitaxel exerts an anti-proliferative effect by interfering with cell microtubule function [18]. Systemic administration of paclitaxel after angioplasty in the rat carotid artery showed that a significant reduction in neointimal proliferation could be achieved at doses much lower than antineoplastic levels [19]. In rat and human cultured cell models, paclitaxel inhibited vascular smooth muscle cell migration and proliferation [19, 20], consistent with its effects in vivo. As an alternative to systemic therapy, local drug delivery offers the advantages of allowing high local concentrations of drug at the treatment site while minimising systemic toxic effects. Proof of this possibility was initially shown using paclitaxel-coated stents in pig coronary arteries [21].

Recent advances in technology have allowed angioplasty balloons to be coated with paclitaxel. This allows local delivery of paclitaxel to the site of stenosis. A number of multicentre, randomised controlled trials (RCTs) in the coronary and peripheral arterial circulation have established the positive benefit of drug-coated balloons (DCBs) [22, 23]. A small pilot study has suggested efficacy in dialysis patients [24, 25]. In this study, 40 patients with dysfunctional AVFs or AVGs were randomised to receive either DCB or plain balloon angioplasty (PBA). Primary unassisted patency (defined angiographically as a binary readout of less than 50 % stenosis) in the DCB group was significantly better than the PBA group at 6 (70 % versus 25 %) and 12 months (35 % versus 5 %, *p* <0.001) respectively. This study may be criticised on a number of points. These include the use of an angiographic rather than a clinical endpoint, the lack of blinding and independent angiographic core laboratory analysis and the very small sample size originally intended to test non-inferiority only (with a wide 15 % non-inferiority limit). In addition, a range of balloons was used in the control group for post dilatation after the paclitaxel-coated balloons, and these were not universally high pressure and non-compliant. This may have added variability to the outcome. Furthermore, the inclusion of both AVFs (35 %) and AVGs (65 %) may have resulted in significant confounding given the difference in survival rates associated to the two types of access. Despite these limitations, the results suggested that a further study of efficacy is warranted, which is what we propose here.

The PAVE trial is a large-scale RCT designed to test superiority of DCBs in native haemodialysis access circuits. Further, the impact on patient quality of life will be assessed. Patient blood samples will also be collected within the setting of the clinical trial. This will form an important resource for future laboratory-based studies on biomarkers and AVF outcomes.

## Methods/design

### Trial objectives

The purpose of this RCT is to compare the efficacy of additional paclitaxel-coated balloon fistuloplasty versus plain balloon fistuloplasty only to preserve the patency of arteriovenous fistulae used for haemodialysis.

### Primary endpoint

#### Time to end of target lesion primary patency

This is defined as patency with no re-intervention to the area 5 mm proximal to, within, and 5 mm distal to, the index treatment segment. Target lesion primary patency ends when *any* of the following occur: (1) clinically driven re-intervention to the treatment segment, (2) thrombotic occlusion that includes the treatment segment, (3) surgical intervention that excludes the treatment segment from the access circuit, (4) abandonment of the AVF due to an inability to re-treat the treatment segment.

In order to confirm that there is a significant stenosis prior to fistuloplasty, duplex ultrasound is encouraged but is not mandatory. It is not mandatory as patients are not randomised until eligibility is angiographically confirmed.

After the study treatment, occasionally there may be recoil or rupture necessitating further balloon angioplasty or stent placement. Providing further angioplasty and/or stent placement achieves a residual stenosis of less than 30 %, these patients will remain in the study.

Referral for a repeat procedure will originate from the clinical team who are unaware of whether the patient received treatment with a paclitaxel-coated balloon or uncoated control balloon.

A different radiologist to the one performing the index procedure will perform repeat procedures when possible but it is not possible to guarantee this. Therefore, the radiologist performing the repeat procedure may have knowledge of whether the patient was treated with DCB or placebo.

In order to ensure that there is no bias in the final decision to proceed with the repeat intervention in patients who have not yet reached the primary endpoint, any pre-procedure fistulogram prior to potential re-intervention will undergo independent core laboratory analysis. This will allow confirmation that a significant stenosis was found in all patients who received a repeat intervention.

### Secondary endpoints

1. Angiographically determined late lumen loss.
  This is the difference between the diameter of the treatment segment post procedure and the diameter at 6 months as measured by an independent core laboratory. If a patient has a repeat procedure to the treatment segment before 6 months, then the pre-intervention images will be used for analysis and a fistulogram at 6 months will not be performed

2. The rate of angiographic binary re-stenosis.
  This is defined as the incidence of stenosis of at least 50 % within the treated lesion at the 6-month follow-up fistulogram. If a patient has a repeat procedure to the index lesion before 6 months, then the pre-intervention images will be used for analysis and a fistulogram at 6 months will not be performed

3. Time to end of access circuit primary patency.
  The access circuit is defined as starting at the arterial anastomosis and ending at the cavoatrial junction. Access circuit primary patency ends when *any* of the following occur: (1) access circuit thrombosis, (2) an intervention (either radiological or surgical) anywhere in the access circuit, or (3) the access circuit is abandoned due to an inability to treat any lesion

4. Time to end of access circuit cumulative patency.
  Access circuit cumulative patency ends when the AVF is abandoned, regardless of radiological or surgical intervention, with or without a thrombotic event. Multiple/repetitive treatments for stenoses that restore patency are compatible with cumulative patency

5. Procedural success (residual stenosis of 30 % or less on completion fistulogram II, after the study treatment)
6. Number of thrombotic events
7. Total number of interventions
8. Adverse events (e.g. fistula rupture, infection)
9. Patient quality of life as assessed by the EuroQol EQ-5D generic health survey, and the disease-specific Patient (or Palliative care) Outcome Scale symptom score-renal (POS-S Renal) [26].

### Trial design

**Fig. 1:**
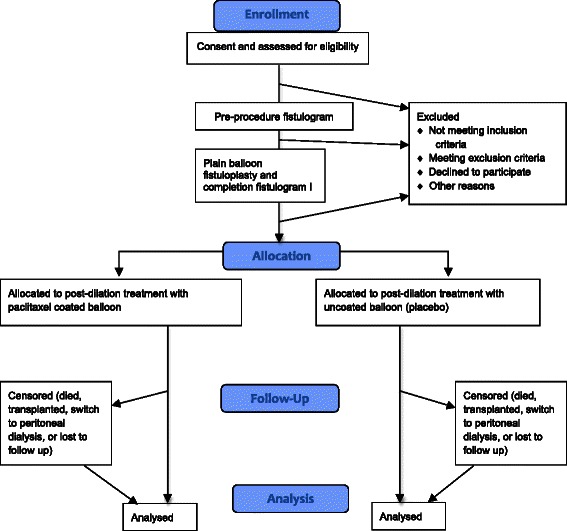
Trial flow chart showing patient progression through the study

The study design used to achieve this will be a multicentre, double-blind RCT. We will recruit 211 patients over a 2-year period from an anticipated six centres. We do not anticipate any differences in relevant clinical characteristics between centres. However, the level of difference will be tested at the analysis stage and trial centre will, therefore, be included as a possible covariate in the primary analysis model. Patients will be followed up for a minimum of 1 year, and all patients will continue in the study until the last patient has completed 1 year of follow-up. Figure 1 shows the trial flow chart.

### Trial statistics

#### Analysis of primary outcome

To test the superiority of the paclitaxel-coated balloon treatment group compared to placebo balloon in target lesion primary patency (TLPP) survival we will use Cox proportional hazards regression, on an intention-to-treat basis. Primary analysis will be repeated using multivariate Cox regression for the adjustment of the treatment effect size for the effect of known clinical covariates and minimisation factors. Model components considered in the primary model will include previous radiological intervention in the access circuit; trial centre; whether the patient is on haemodialysis or not at study entry; trial arm; observed study time (length of time between patient entering and exiting study); and a trial arm × observed time interaction term. The interaction term allows for variable follow-up time effects. Patients with TLPP at the end of follow-up will be considered censored, as will those who receive a renal transplant, switch to peritoneal dialysis or are lost to follow-up before the study end. Kaplan-Meier plots, hazard ratio (HR) and its confidence interval will be used to describe the results.

#### Analysis of secondary outcomes

Effects on secondary outcomes will be analysed using the same strategy for time-to-event variables, and generalised linear models for binary and continuous outcome measures, adjusting for the effects of relevant covariates when appropriate. Continuous variables will be checked for normality, transformed if necessary or otherwise analysed using a Wilcoxon signed-rank test for independent samples.

#### Missing values and drop-outs

Withdrawal from trial follow-up (attrition rate) will be reported by intervention group, including reasons for withdrawal. The proportions of participants missing each variable will be summarised in each arm and at each study visit. If necessary, multiple imputation will be used for the imputation of missing values in baseline variables and secondary outcomes. Patients with TLPP at the end of follow-up will be considered censored, as will those who receive a renal transplant, switch to peritoneal dialysis or are lost to follow-up before the study end. The baseline characteristics and adverse events (AEs) of patients lost to follow-up will be compared to those with complete follow-up data. The relationship between these and missing data will be investigated graphically to see if baseline characteristics or AEs predict missing values, i.e. drop-outs are not random. Should any baseline variables be predictive of missing values then these will be included in the primary analysis Cox regression models as further covariates.

For non-time-to-event outcomes, missing post-randomisation assessments will be dealt with by fitting generalised linear models to all the available data using maximum likelihood methods. Such an approach provides valid inferences under the assumption that the missing data mechanism is ignorable (or missing at random (MAR)). This allows for missingness at later times to be predicted by outcome values at earlier times. However, if post-treatment variables, such as compliance with study procedures, are found to be predictive of drop-out, multiple imputation will be considered.

#### Interim analysis

Interim analysis of the primary outcome will be performed three times throughout the study based on the cumulative number of failures of the treatment area, i.e. after 27, 54 and 81 events, expected approximately at 9, 14 and 19 months of study under the null hypothesis, and at months 11, 17, and 23 under the alternative hypothesis. Group sequential stopping boundaries have been calculated using a Lan-DeMets spending function (with O’Brien-Fleming parameters), to allow early stopping for rejection of the null or the alternative hypotheses. Stopping in case of boundary crossing is non-binding.

#### Model assumption checks

In order to assess the adequacy of the Cox regression models for the primary outcome and time-to-event secondary outcomes, the main assumption to test for is proportionality; the Kaplan-Meier plots will be used to check if the curves for the two trial arms are the same shape, and if the separation of the curves remains proportionate throughout the analysis period. In addition, time-dependent covariates will be generated by creating interactions of the predictors and function of survival time; if these are significant then the predictors are not proportional. If the assumption for proportionality is violated then the consequence this has on the results can be checked. The Cox model can be stratified according to the variables with non-proportional hazards to see whether this changes the HRs for the variables of interest; if it still does, then it may be necessary to use an alternative model. One parametric alternative is the Royston-Parmar model, which is more flexible and can fit a non-proportional hazards model. For the other secondary outcomes regression residuals will be plotted to check for normality and outliers, where applicable.

### Sample size, selection and withdrawal of subjects

For the definition of the survival curve in the placebo balloon group, we assumed target lesion primary patency rates of 61 %, 42 %, and 35 % at 6, 12 and 24 months respectively. This was consistent with published results [7] and with our own audit data. A HR of 0.5 was chosen as the minimum clinically relevant effect size. Katsanos et al. [24] found a HR of 0.3 for TLPP at 6 months; however, the confidence interval was broad and the effect size is expected to be closer to the null when AVGs are excluded. Based on these assumptions, it is expected that the paclitaxel-coated balloon group will show 78 %, 65 %, and 59 % survival rates of TLPP at 6, 12 and 24 months respectively. Recruiting 211 patients, with variable follow-up, a minimum follow-up of 1 year, and three interim analyses, will provide 94 % power to detect a statistically significant difference between the two groups in TLPP survival rates with a two-sided 5 % type I error rate. It is expected that 108 patients will experience fistula failure during the follow-up period.

The required sample size has been estimated assuming cumulative 10 % drop-out in each treatment arm by the end of the study, and recruitment of 2 patients per month (ppm) during the first 3 months, 8 ppm up to 7 months, and 12 ppm onwards. The expected accrual duration will be 22 months, and the maximum study duration (including follow-up) 34 months. We do not anticipate patients completely withdrawing from the trial as the primary endpoint data are collected primarily from clinical records. The 10 % drop-out estimate is based primarily on switching to peritoneal dialysis, receiving a transplant, or death.

The inclusion and exclusion criteria are summarised in Table 1.

**Table 1:** Inclusion and exclusion criteria for the trial

Inclusion criteria	Exclusion criteria
1. Patients (18 years or over) who have a native AVF in the arm	1. Patient unable to give informed consent
2. An indication for a fistuloplasty as determined by the local clinical team	2. Patient unwilling or unable to comply with all study-related procedures
3. The access circuit is free of synthetic graft material or stents	3. Systemic or local (to the fistula) infection treated for less than 10 days prior to the study procedure
4. A reduction of vessel diameter ≥50 % measured angiographically and reference diameter of the outflow vein ≥4 mm and less than the size of the largest available DCB	4. Synchronous venous lesion, with a reduction of vessel diameter of ≥50 % measured angiographically, in the same access circuit
5. A residual stenosis ≤30 % after plain balloon fistuloplasty	5. Location of stenosis central to the thoracic inlet
	6. Thrombosed (failed) access circuit at time of treatment
	7. Women who are breastfeeding, pregnant or are intending to become pregnant or men intending to father children, within 2 years of study treatment
	8. Known hypersensitivity or contraindication to contrast medium which cannot be adequately premedicated
	9. Known hypersensitivity or contraindication to paclitaxel

*AVF* arteriovenous fistula, *DCB* drug-coated balloon

### Criteria for premature withdrawal

Participants have the right to withdraw from the study at any time for any reason.

Participants will be withdrawn from the study if any of the following occur:

- Death of participant
- Participant receives a transplant
- Participant is changed from haemodialysis to peritoneal dialysis

The principal investigator (PI) also has the right to withdraw patients from the study in the event of inter-current illness, AEs, serious adverse events (SAEs), protocol violations, administrative reasons or other reasons, e.g. the participant is no longer being treated at a hospital included in the study.

It is understood by all concerned that an excessive rate of withdrawals can render the study uninterpretable; therefore, unnecessary withdrawal of patients should be avoided. Should a patient decide to withdraw from the study, all efforts will be made to report the reason for withdrawal as thoroughly as possible. Participants who wish to withdraw from ‘treatment’ will be asked to confirm whether they are still willing to provide study-specific data and samples for scientific laboratory analysis according to the trial protocol.

### Screening procedures

Patients who may be eligible will be identified in a vascular access clinic and assessed by surgeons, specialist nurses and nephrologists.

In order to confirm that there is a significant stenosis prior to angiography, a duplex ultrasound is encouraged but is not mandatory.

At least 24 hours after being given the patient information sheet and before entering the interventional suite, consent will be taken and eligibility criteria as listed above in Table 1 will be assessed. Inclusion criteria 1–3 will be confirmed and exclusion criteria 1–3 and 6–9 will be assessed.

The radiologist who will perform the procedure will be informed that the patient is potentially eligible for the study.

All procedures will be performed in a dedicated interventional radiology suite equipped with digital subtraction angiogram, image overlay/roadmap post-processing capabilities and ability to capture still and video DICOM file data.

### The pre-procedure fistulogram

This will be take place immediately prior to the plain balloon fistuloplasty. It will be performed through a sheath or cannula placed in the dialysis circuit according to the specifications summarised in Table 2.

**Table 2:** Specifications for the pre-procedure fistulogram and the 6-month protocol fistulogram

It will be performed in a dedicated interventional radiology suite equipped with digital subtraction angiogram, image overlay/roadmap post-processing capabilities and ability to capture still and video DICOM file data.
It will be performed through a sheath or cannula placed in the dialysis circuit according to the following specifications:
1. All fistulograms performed as digital subtraction acquisitions at three frames per second
2. The entire access circuit from anastomosis to central vein covered in up to three stages
3. Medial epicondyle of humerus as visible bony landmark on lower arm acquisition; acromioclavicular joint on upper arm and central acquisitions
4. Measurement ruler in view
5. Lower arm acquisition to include:
(a) Anteroposterior projection of anastomosis
(b) Oblique projection of anastomosis (specify oblique and craniocaudal angulation)
6. On the acquisition that best demonstrates the target lesion, the following measurements are made:
(a) Peripheral (close to anastomosis) reference vessel diameter
(b) Minimum lumen diameter (MLD)
(c) Central reference vessel diameter

Specifications for (a) the pre-procedure fistulogram, (b) the 6-month protocol fistulogram, and (c) fistulograms performed for a clinical indication in patients who have: (i) not yet reached the primary endpoint, and (ii) are referred for a potential re-intervention by the radiologist who performed the study treatment and so is not blinded to this

The radiologist will assess inclusion criteria 3 and 4, and exclusion criteria 4 and 5, to decide if the patient remains eligible for the study.

### The plain balloon fistuloplasty procedure

Prior to treatment 3000–5000 IU of heparin is administered. For all patients treatment has two components. The fistuloplasty procedure is performed with a dedicated plain balloon (Bard Dorado) ensuring the following criteria are met:

1. Sized to nominal vein diameter
2. Up to 24 Atm of pressure to ensure obliteration of the lesion waist
3. Minimum duration of balloon inflation of 1 minute

If further plain balloon fistuloplasty treatment is required, then this may be administered twice more only prior to the study treatment.

Completion fistulogram I is performed after the plain balloon fistuloplasty to ensure adequate therapy according to the specifications in Table 3.

**Table 3:** Specifications for completion fistulograms I and II

1. All fistulograms performed as digital subtraction acquisitions at three frames per second
2. Acquisition that demonstrates the target lesion is matched as close as possible to the respective pre-procedure fistulogram acquisition
3. Measurement ruler in view
4. The following measurements are made:
(a) Peripheral (close to anastomosis) reference vessel diameter
(b) Minimum lumen diameter (MLD)
(c) Central reference vessel diameter

The radiologist will assess completion fistulogram I and decide if the residual stenosis is 30 % or less (inclusion criteria 5). If this is the case the patient will proceed to randomisation, and if not the patient will be excluded.

### Randomisation procedures

Randomisation will be at the level of the individual participants, minimising on the radiologist performing the study procedure, whether the participant is currently on haemodialysis or not, and whether the participant has had a previous radiological intervention in the access circuit or not. This is performed with an 80 % probability of allocating to the arm which reduces the imbalance. The allocation sequence will be generated dynamically. This way, the next allocation will only be generated and become known upon actioning a request from the study site staff.

Minimisation will be implemented using an independent web-based randomisation system hosted at the UKCRC-registered clinical trials unit at KCL. Site staff will access the service via www.ctu.co.uk using a computer in the angiography room or an office nearby. It will be performed by the radiologist or their nominee who will log into the system, enter the participant ID number, initials, date of birth, recruiting radiologist, whether the participant is currently on haemodialysis or not, and whether the participant has had a previous radiological intervention in the access circuit or not. Nominees must not be clinicians or nurses who may decide to refer the patient for re-intervention. Each randomiser will have a unique user access, provided by the Clinical Trials Unit (CTU) upon the authorisation of the trial manager, once the delegation of authority form has been completed.

Once randomised, the system will automatically generate a confirmation email, which will be sent to relevant study staff in a blinded or unblinded format, depending on their role in the study.

If it is not possible to use the randomisation system randomisation may occur using the toss of a coin in order to avoid losing the patient from the study. *This should only be needed, if at all, in specific and rare situations such as the CTU server being inaccessible.* This will be performed by two people with heads denoting DCB, and tails denoting placebo. The CTU must be informed of the coin randomisation as soon as possible.

### Study treatment

In the intervention arm, the second component is insertion of a DCB (Bard Lutonix). This must be of identical diameter to the plain balloon (Bard Dorado) and a minimum of 1 cm longer than the plain balloon (Bard Dorado) (5 mm at either end), inflated to nominal pressure at the lesion location for a minimum duration of 1 minute.

Instructions for use of the DCB are stringently adhered to to ensure appropriate preparation and handling of the device.

In the control arm, an identical procedure is followed, but using a placebo balloon that is not drug-coated (Bard Ultraverse). This must be of identical diameter to the plain balloon (Bard Dorado) and a minimum of 1 cm longer than the plain balloon (Bard Dorado) (5 mm at either end), inflated to nominal pressure at the lesion location for a minimum duration of 1 minute.

If more than one plain balloon (Bard Dorado) is used for the plain balloon fistuloplasty then the dimensions of the placebo balloon (Bard Ultraverse) *or* drug-coated balloon (Bard Lutonix) is matched to the plain balloon with the larger diameter and/or the longer length.

In both arms, image overlay/roadmap will be utilised to ensure that there is no geographical mismatch between the segments treated with the high- and low-pressure balloons.

A completion fistulogram is performed (completion fistulogram II) to confirm that there is no angiographically visible effect after treatment with the drug-coated or placebo balloon, according to the same specifications as fistulogram I in Table 3. Procedural success is defined as a residual stenosis of 30 % or less on completion fistulogram II.

The data file(s) containing the initial pre-procedure fistulogram and completion fistulograms I and II will be sent to the lead study site with the patient’s name replaced by the trial ID, and with each of the above groups of images clearly identified. Completion fistulogram II will then be sent to the independent angiographic laboratory for analysis.

### Study assessments

These will occur every 3 months ± 1 month. Follow-up will be variable but for a minimum of 1 year and a maximum of 3 years. These will involve a clinical assessment to take place either face to face or via a telephone conversation. Any face-to-face meetings will usually coincide with dialysis to avoid additional patient travel.

Data recorded for each study assessment will include the following: target lesion primary patency, access circuit primary and cumulative patency, access circuit interventions, patient medications and AEs.

At the 6-month study assessment, the trial team will additionally collect information on fistula function and check if referral for re-intervention is being considered based on clinical concerns. If this is the case then a fistulogram ± plasty will be performed according to usual clinical practice and the patient will not undergo a protocol fistulogram. If there are no clinical concerns related to the fistula, then patients will be invited to undergo a protocol fistulogram. Confirmation that there is no contraindication to this protocol fistulogram will be obtained from an appropriate doctor and documented.

### Six-month protocol fistulogram

This will take place within 2 weeks of the 6-month study assessment. If a patient has required a repeat fistuloplasty to the treatment segment at or before 6 months then they will not undergo the 6-month protocol fistulogram. All other patients will be invited to undergo a protocol fistulogram 6 months after the index procedure to acquire the data for the angiographic secondary endpoints. If a patient declines the 6-month protocol fistulogram or does not have it for another reason, this will not be considered a protocol violation and the patient may continue in the study.

The 6-month protocol fistulogram *must* be performed by a radiologist other than the one who performed the index procedure to ensure that they are blind to which trial arm the participant belongs. With forward planning this should be possible, but if it is not then the protocol fistulogram should not be performed.

The 6-month protocol fistulogram will be a diagnostic study only unless an unsuspected stenosis is identified and the radiologist believes that it would be unethical not to intervene. This will not be considered a protocol violation and a fistula intervention form will need to be completed.

The 6-month protocol fistulogram will be performed according to the specifications in Table 2.

The 6-month protocol fistulogram will be considered to be exclusively trial data. The result of the 6-month protocol fistulogram will not be made available (verbally or in writing) to the clinical team responsible for considering future referral of the patient for an intervention. The images will also not be available on the local radiology system. The images will be sent to the lead site in order to be forwarded to the independent core laboratory with the patient’s name replaced by the trial ID.

### Fistulograms performed for a clinical indication

The following applies only to patients who have not yet reached the primary endpoint of the trial, and who are referred for a potential re-intervention by the radiologist who performed the study treatment and so is not blinded to this. Pre-procedure fistulograms performed for a clinical indication will follow the specifications in Table 2.

The image file will be sent to the lead site in order to be forwarded to the independent core laboratory for analysis with the patient details replaced by the trial PIN. This will be sent regardless of whether the fistulogram is followed by a fistuloplasty. This will allow us to demonstrate that there is no bias in the final decision to proceed, or not, with the repeat intervention.

### End of study definition

The clinical trial will end when 211 patients have been recruited and all patients have completed at least 1 year of follow-up. The trial may be prematurely discontinued by the sponsor, funder, chief investigator or Trial Steering Committee (TSC) on the basis of new safety information or for other reasons given by the Data Monitoring and Ethics Committee (DMC), the TSC, and the Research and Ethics Committee (REC). The trial may also be prematurely discontinued due to lack of recruitment or upon advice from the TSC which will advise on whether to continue or discontinue the study and make a recommendation to the sponsor. If the trial is prematurely discontinued, active participants will be informed and no further participant data will be collected.

### Laboratory tests

There are no local laboratory tests that are required to provide data that directly relate to trial endpoints. A 10-ml blood sample will be requested at the time points stated in the trial schedule, and is to be sent to the local clinical laboratory for a full blood count and to check the C-reactive protein level. If patients decline some or all of these samples it will not be considered a protocol violation.

Blood (up to 90 ml) may be taken at each of the time points. These will be sent to the research laboratory of the chief investigator (CI) where the blood will be separated. Research blood samples should not be taken from patients who are known to be hepatitis B surface antigen- (HbsAg), hepatitis C IgG/RNA- or HIV-positive. Deoxyribonucleic acid (DNA) and ribonucleic acid (RNA) will be stored. Cells will be stored in aliquots in liquid nitrogen until thawed for analysis. Serum and/or plasma samples will be stored at −20 °C or −80 °C until thawed for analysis. Transport, separation and storage will be according to Standard Operating Procedures. It will not be a considered a protocol violation if any of the blood samples are not taken, or are taken at different time points to those specified and patients may continue in the study.

### Independent core laboratory analysis

The completion fistulogram II (taken after treatment with the DCB or placebo low-pressure balloon) will be compared with the protocol 6-month fistulogram or with the pre-procedure fistulogram taken prior to a clinically driven re-intervention at the treatment segment if this is before 6 months. These will be analysed by an independent core laboratory for the angiographic secondary endpoints.

In addition, in patients who have not yet reached the primary endpoint of the trial, clinically-driven pre-procedure fistulograms will be sent to the independent core laboratory for analysis if they were performed by a radiologist who is not blind to the study treatment. This will be sent regardless of whether the fistulogram is followed by a fistuloplasty.

### Assessment of safety

We have been informed by the Medicines and Healthcare products Regulatory Agency (MHRA) that the PAVE protocol does not fall within the Clinical Trial Regulations and, therefore, is not a drug trial. In addition, the DCB is a CE-marked medical device, so prior regulatory approval from the MHRA is not needed. Safety reporting will be in keeping with the requirements for research other than Clinical Trials of Investigational Medicinal Products.

### Data Monitoring and Trial Steering Committees

The membership will be decided by the CI and approved by the National Institute for Health Research (NIHR). The DMC includes a statistician and two other independent experts. They will receive a report of recruitment, serious and non-serious adverse events and a summary of accumulated clinical data from the trial statistician, and will meet in person or by telephone. They will report to the TSC who will usually meet in the 2 weeks following the DMC meeting. The DMC will meet at least annually during the study, approximately 2 weeks prior to the TSC. Additional meetings may take place at the time of interim analysis or in case of recruitment issues. The DMC is advisory to the TSC. The DMC charter will be drafted and agreed prior to recruitment. The trial statistician will prepare reports to the DMC. The TSC will be convened in the post-award period. The membership will be decided by the CI and approved by the NIHR. The chair will be an independent expert. Members will include the CI, a patient representative, and two other independent experts. The TSC will meet at least annually during the study, approximately 2 weeks after the DMC. Additional meetings may take place at the time of interim analysis or in case of recruitment issues. The TSC is an executive committee. Terms of reference of the TSC will be agreed and documented prior to start of recruitment. The trial manager will prepare reports to the TSC.

### Ethics and regulatory approvals

Informed consent will be obtained from all participants. The trial will be conducted in compliance with the principles of the Declaration of Helsinki (1996), the principles of GCP and in accordance with all applicable regulatory requirements including but not limited to the Research Governance Framework. This protocol and related documents were approved by the London-Chelsea REC on 12 May 2015 (reference 15/LO/0638). The CI will submit a final report to the sponsor and the REC at conclusion of the trial. Annual progress reports will be submitted to the main REC for the study.

### Data handling

All samples will be anonymised before laboratory analysis. No patient-related data will be held in research laboratories. During the study, any paper documents will be held in a locked filing cabinet in a locked office and retained for a minimum of 5 years following the end of the study. Clinical and research data for the study will be stored on the eCRF system, hosted at the King’s College London Clinical Trials Unit. The eCRF (InferMed MACRO) is GCP and FDA 21 CFR Part 11 compliant. Data entry staff at site will be provided with unique usernames and passwords to the system and will be trained in data entry by the trial manager. The trial manager will visit sites to review data on the system, raise discrepancies and confirm source data verification checks. All requests for access to the data entry system must be authorised by the trial manager. All requests for data exports must be authorised by the trial statistician. The trial manager will work with the CI and the trial statistician to ensure that data are checked and cleaned on an ongoing basis and will confirm that all data checks have been completed before database lock. The investigators and the institutions will permit trial-related monitoring, audits, REC review, and regulatory inspections (where appropriate) by providing direct access to source data and other relevant documents (i.e. patients’ case sheets, blood test reports, X-ray reports). Record keeping will be the responsibility of the investigators.

## Discussion

We have presented the protocol of a multicentre RCT to test whether use of DCBs will lead to preservation of vascular access for haemodialysis with a reduction in restenosis and the need for repeat fistuloplasties. There is one previously published randomised trial in vascular access for haemodialysis patients and the limitations of this study have been discussed above in the Background section [24]. Two recent observational reports give further support to the suggestion that DCBs will be of benefit. A single-centre study investigated DCBs for the management of 20 juxta-anastomotic stenoses in radiocephalic fistulae of 10 patients. A different lesion in the same patient served as the control [27]. This small study suggested an improved target lesion revascularisation-free survival for DCBs. In addition, in a retrospective study of recurrent symptomatic central stenosis in 27 consecutive patients with haemodialysis fistulas, custom-made DCBs were suggested to show a benefit over plain balloons [28].

We are aware of two prospective randomised trials that have been completed with results awaited. One is a single centre study from Singapore (NCT01544907) and includes 125 patients. It includes a mixture of AVGs and AVFs and has a primary endpoint of angiographic late lumen loss. In addition, a small randomised open-label study from Canada (NCT01001676) includes 33 patients with AVGs or AVFs and has 6-month primary patency as the primary endpoint.

In contrast to these trials, our study is restricted to patients with a native AVF in order to give a more defined patient group. In addition, our trial design means that patients and referring clinicians will be blinded to the study treatment. Furthermore, the primary endpoint is a clinical one that makes a difference to patients. We have included two secondary angiographic endpoints, which will be measured during a protocol fistulogram at 6 months post randomisation in patients who have not met the primary endpoint prior to this. We anticipate that the number of patients who will have had a repeat procedure within these 6 months will be fewer in the DCB treatment group compared to the control group. This means that the timing of the fistulogram used for the secondary angiographic endpoints will, on average, be later for the DCB treatment group. Therefore, any improvement in late lumen loss or binary restenosis in the DCB group will be despite this difference in timing, rather than because of it. We anticipate that this trial will provide rigorous data that will clarify whether DCBs are of clinical benefit in haemodialysis patients with a native AVF undergoing fistuloplasty.

### Trial status

Recruitment started in November 2015.

## Abbreviations

AE: adverse event
AR: adverse reaction
AVF: arteriovenous fistula
AVG: arteriovenous graft
Atm: atmospheres (pressure)
CI: chief investigator
DCB: drug-coated balloon
DMC: Data Monitoring Committee
PI: principle investigator
PTA: percutaneous transluminal angioplasty
RCT: randomised controlled trial
SAE: serious adverse event
TLPP: target lesion primary patency
TSC: Trial Steering Committee
